# Case Report: Aicardi-Goutières Syndrome and Singleton-Merten Syndrome Caused by a Gain-of-Function Mutation in IFIH1

**DOI:** 10.3389/fgene.2021.660953

**Published:** 2021-05-13

**Authors:** Wei Xiao, Jie Feng, Hongyu Long, Bo Xiao, Zhaohui H. Luo

**Affiliations:** Department of Neurology, Xiangya Hospital, Central South University, Changsha, China

**Keywords:** Aicardi-Goutières syndrome, Singleton-Merten syndrome, IFIH1, type I IFN, autoimmunity

## Abstract

The *IFIH1* gene encodes melanoma differentiation-associated gene 5 (MDA5) and has been associated with Aicardi-Goutières syndrome (AGS), Singleton-Merten syndrome (SMS), and other autoimmune diseases. The mechanisms responsible for how a functional change in a single gene can cause so many different phenotypes remain unknown. Moreover, there is significant controversy as to whether these distinct phenotypes represent the same disease continuum or mutation-specific disorders. Here, we describe the case of a patient with a novel c.1465G > T (p.Ala489Ser) mutation in the *IFIH1* gene. The patient presented with spastic paraplegia, dystonia, psychomotor retardation, joint deformities, intracranial calcification, abnormal dentition, characteristic facial features, lymphadenopathy, and autoimmunity. His phenotype appeared to represent an overlap of the phenotypes for AGS and SMS. The patient also experienced unexplained pancytopenia, suggesting that the hemic system may have been affected by a gain-of-function mutation in the *IFIH1* gene. In summary, we provide further evidence that SMS and AGS exhibit the same disease spectrum following a gain-of-function mutation in the *IFIH1* gene. Our data highlight the genetic heterogeneity of these conditions and expand our knowledge of differential phenotypes created by *IFIH1* gain-of-function mutation.

## Introduction

The IFIH1 gene encodes melanoma differentiation-associated gene 5 (MDA5), a cytosolic double-stranded RNA sensor. IFIH1 gain-of-function mutations lead to the inappropriate sensing of self-derived nucleic acid as a viral product, thus causing an aberrant interferon (IFN) response, leading to extensive organ damage (Ahmad et al., 2018). Aicardi-Goutières syndrome (AGS) is a genetic autoimmune disorder that exerts particular influence on the skin and brain (Orcesi et al., 2009); the main pathophysiological feature of this condition is the overproduction of type I IFN (Chahwan and Chahwan, 2012). Until now, specific mutations in seven different genes have been reported to be associated with AGS, including RNASEH2A, TREX1, ADAR, RNASEH2C, RNASEH2B, SAMHD1, and IFIH1 (Crow and Manel, 2015). Singleton-Merten syndrome (SMS) manifests as aortic calcification, dental dysplasia, and skeletal abnormalities (Gay and Kuhn, 1976). Although this appears to be a clinically distinct condition, a substitution mutation (p.Arg822Gln) in the IFIH1 gene has also been identified in SMS (Rutsch et al., 2015). Previous reports have described patients exhibiting c.1465G > A and c.992C > T mutations in the *IFIH1* gene that presented with phenotypes that appeared to overlap between the typical intracranial calcification of AGS and the clinical manifestation of SMS (Bursztejn et al., 2015; de Carvalho et al., 2017). However, most of these patient did not involve neurological dysfunction.

In this case report, we describe a patient who possessed a c.1465G > T mutation in the IFIH1 gene; he expressed high levels of interferon-stimulated genes. The patient had severe neurological dysfunction. Furthermore, the phenotypic overlap between AGS and SMS was very obvious in this patient. Our case provides further evidence for a phenotypic continuum between AGS and SMS for gain-of-function mutations in the *IFIH1* gene.

## Case Presentation

### Clinical Findings

**Figure 1:**
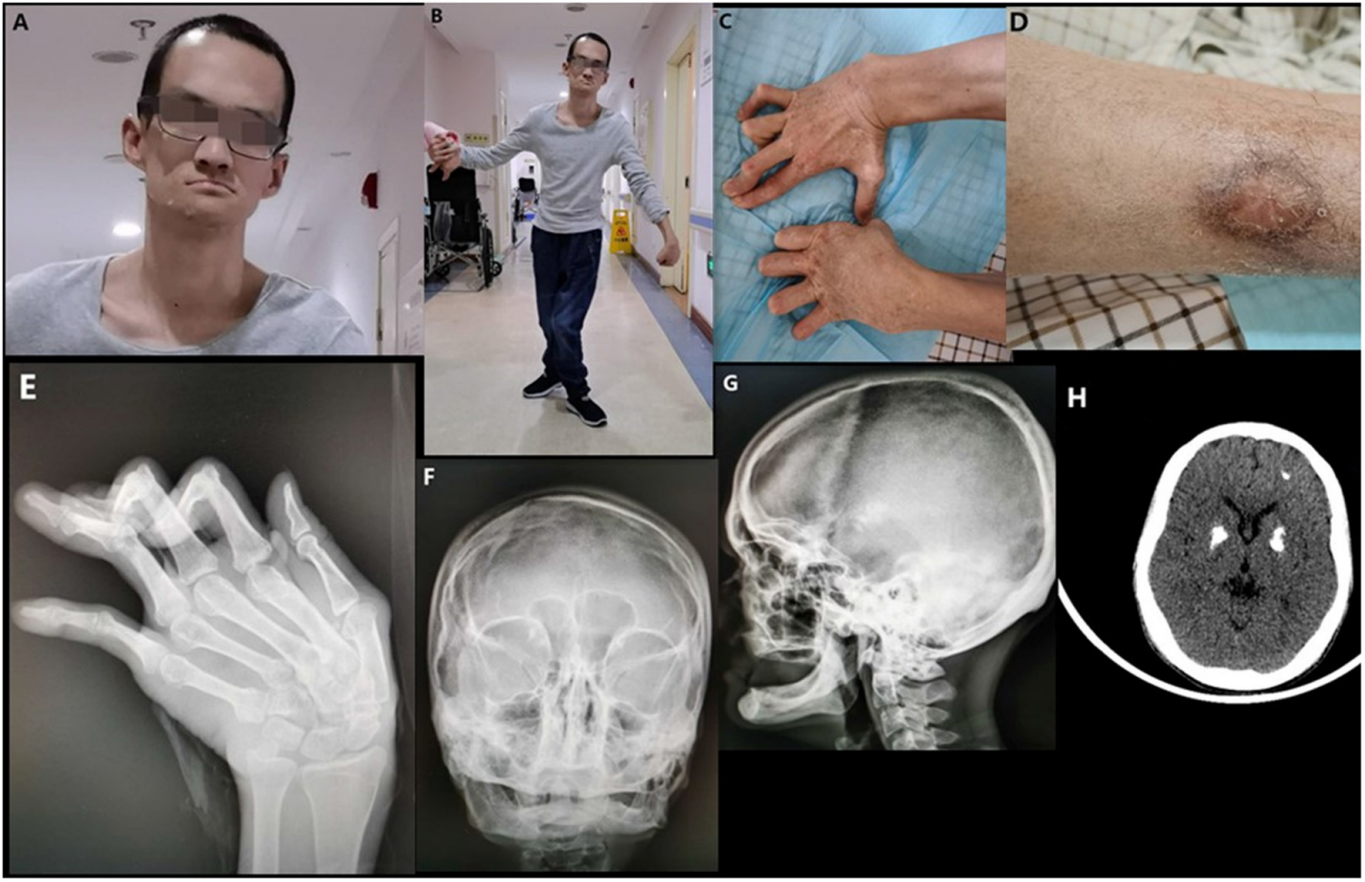
Clinical images of the proband. **(A)** The facial characteristics of the proband (broad forehead, high anterior hairline, and a thin upper vermilion). **(B)** Spastic gait and dystonia of wrists. **(C)** Contracture of the fingers. **(D)** Scarring and pigmentation following ulceration of the leg. **(E)** Deformities of the interphalangeal joints and ulnar deviation of the left metacarpal. **(F)** Deformity of both maxilla and mandible. **(G)** Complete loss of teeth and alveolar bone. **(H)** Multiple intracranial calcifications (bilateral lenticular and frontal lobe).

The proband was born to healthy parents after 39 weeks of gestation. The birth weight was 3,500 g and the delivery process went smoothly. Initial development was normal and the patient was able to sit without support at the age of 6 months. Body weight was within the normal range. He has suffered from recurrent respiratory tract infections and unexplained chronic diarrhea since he was 7 months-of age. There had been a delay in motor development since he was 7 months-of age although mental development appeared to be normal. He was unable to roll or stand at the age of 12 months and did not begin walking until he was 3 years-of-age. Due to spasticity and weakness of the lower limbs, he presented with specific gait disturbances: forward leaning of the trunk and tip-toe walking. These gait disturbances did not improve with body growth, although he could look after himself in terms of normal daily life. Intracranial calcification was detected when he was 5 years-of-age and he was clinically diagnosed with Fahr's syndrome. He has experienced urinary incontinence and ulcerations of the leg since he was 10 years-of-age. These ulcerations are worse in the winter but heal with pigmentation in the summer (Figure 1D). His teeth developed normally but began to loosen during his teenage years. Ultimately, the patient had lost all of his adult teeth by the time he was 20 years-of-age (no data were available on his primary dentition). When he was 23 years-of-age, the patient began to experience the progressive but painless contracture of his fingers and developed deformities of the interphalangeal joints (Figure 1C). Owing to pain from his knees and hip joints, the patient was not able to walk independently from the time he was 28 years-of-age. Furthermore, he developed dystonia of the wrists, dysphagia, and dysarthria.

On examination in our hospital, the patient was 30 years-of-age. He had unusual facial features. He had a broad forehead, a high anterior hairline, and a thin upper vermilion (Figure 1A). Neurological examination revealed dystonia of the wrist, a sluggish pharyngeal reflex, and spastic paraplegia of both lower limbs (Figure 1B). X-ray confirmed deformities of the interphalangeal joints of both hands and ulnar deviation of the left metacarpal (Figure 1E). Skull X-rays showed a complete loss of teeth and alveolar jaw bone (Figure 1F). Deformities of the maxilla and mandible were also observed (Figure 1G). Multiple intracranial calcifications were evident on cranial computed tomography (CT) imaging (Figure 1H). Chest CT and sonography of the lymph nodes revealed multiple lymphadenopathy and esophageal hiatal hernia. Cardiac ultrasound was normal and there was no aortic calcification. Numerous blood examinations were performed, including the evaluation of calcium-phosphorus metabolism, immune screening, complement analysis, and inflammatory response. Data revealed normal calcium and phosphorus metabolism, complement activation, and inflammatory response, but abnormal autoantibody profiles and pancytopenia (Table 1).

**Table 1:** Blood analysis of the proband.

**Item**	**Reference**	**The proband**
WBC count (× 10^9^/L)	4–10	3.06↓
Neutrophil count (× 10^9^/L)	2.0–8.0	1.3↓
Hemoglobin (g/L)	130–175	95↓
Platelet (× 10^9^/L)	125–350	82↓
Erythrocyte sedimentation rate (mm/h)	0–21	41↑
C-reactive protein (mg/L)	0–8.00	3.2
C3 (mg/L)	790–1,520	788↓
C4 (mg/L)	100–400	287
Antithyroglobulin antibodies (kU/L)	0–115	296.40↑
Antithyroid peroxidase antibodies (kU/L)	≤ 100	22.96
IgG (gm/L)	5.58–12.54	19.70↑
IgA (gm/L)	0.13–1.08	0.36
IgM (gm/L)	0.4–2.8	0.324↓
ANA titer (normal)	Negative	Negative
Double-stranded DNA (normal)	Negative	Negative
Fecal occult blood test	Negative	Positive

### Molecular Findings

**Figure 2:**
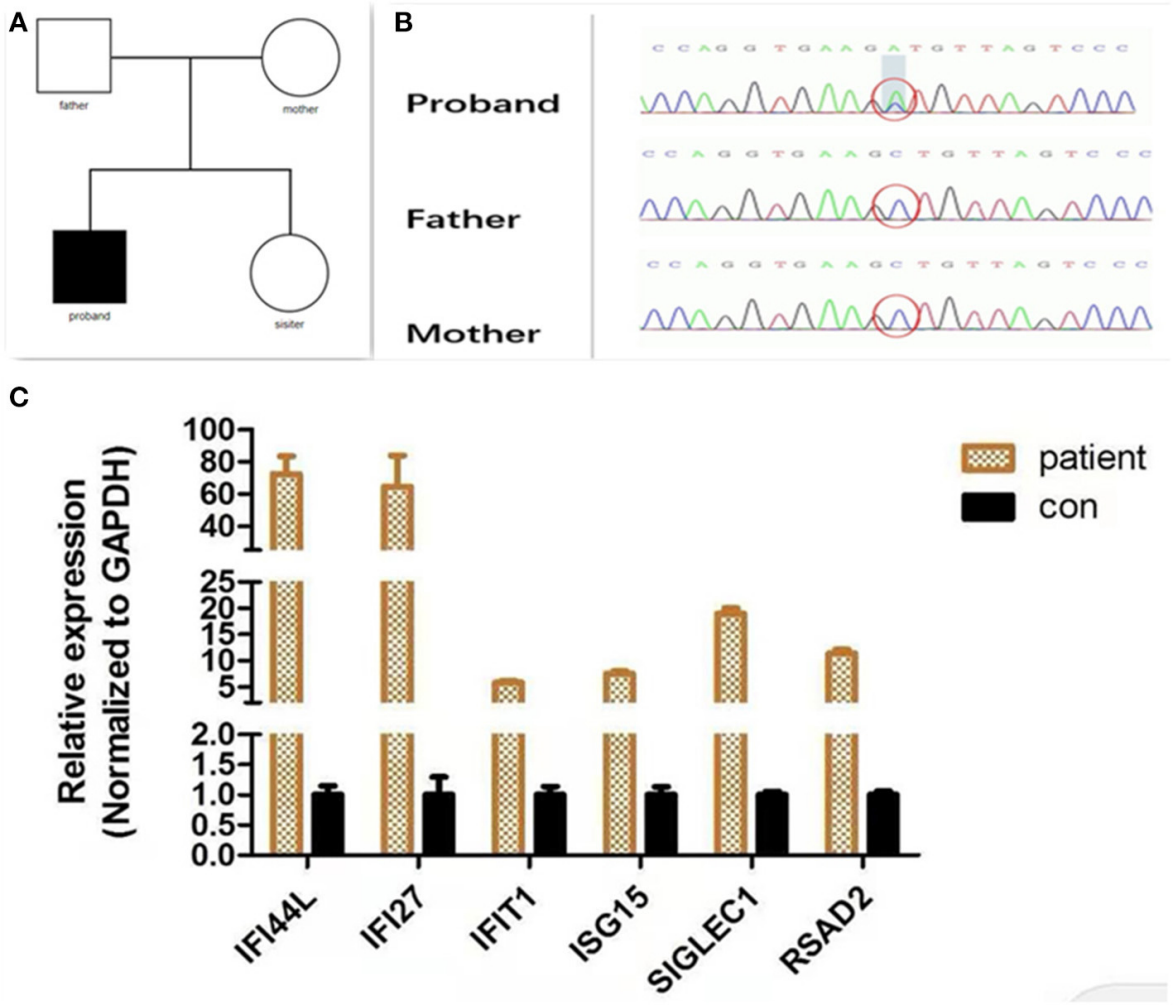
Molecular findings. **(A)** Family tree. **(B)** A *de novo IFIH1* mutation c.1465G > T (p.Ala489Ser) confirmed by Sanger sequencing. **(C)** Quantitative analysis of interferon-stimulated genes. The expression of interferon-stimulated genes (*IFI27, IFIT1, IFI44L, ISG15, SIGLEC1*, and *RSAD2*) was analyzed by quantitative PCR. The relative expression of each interferon-stimulated gene in PBMCs (peripheral blood mononuclear cells) isolated from the patient was normalized to controls and represented as a mean and standard deviation. Control PBMCs were donated from a healthy individual who had been age- and gender-matched. All experiments were carried out in triplicate.

Considering the age at onset and the unusual combination of systemic features, we suspected that a genetic disease was involved and carried out whole exome sequencing (WES). The method of WES was introduced in Supplementary Material. Analysis identified a heterozygous *IFIH1* mutation [c.1465G > T (p.Ala489Ser)] but no other disease-causing genes were detected (Figure 2A). The variant was neither found in Exome Aggregation Consortium (ExAC) data nor Genome Aggregation Database (gnomAD). The pathogenicity of the variants was assessed by four software programs: SIFT/PROVEAN (http://provean.jcvi.org/index.php), PolyPhen2 (www.genetics.bwh.harvard.edu/pph2/), and Mutation Taster (www.mutationtaster.org/). Different web-based prediction tools suggested that the c.1465G > T variant is pathogenic (Supplementary Table 1). Further Sanger sequencing showed that the parents of the proband did not possess this mutation, indicating that this was a *de novo* mutation (Figure 2B). In order to identify an aberrant IFN response, we conducted transcriptional analysis of whole blood RNA by quantitative real-time polymerase chain reaction (qPCR) (The method of qPCR was introduced in Supplementary Material). The proband showed higher interferon-stimulated global gene expression than a healthy control (matched with age and gender) (Figure 2C). Our analysis therefore confirmed that the *IFIH1* mutation [c.1465G > T (p.Ala489Ser)] was pathogenic.

## Discussion

Singleton-Merten syndrome manifests with aortic calcification, dental dysplasia, and skeletal abnormalities, but can also involve additional, less common features, including muscular weakness, psoriasis, and glaucoma (Singleton and Merten, 1973). Our proband presented with dental anomalies, metacarpophalangeal contractures, muscular weakness, deforming arthropathy, an abnormal maxilla, and the complete loss of alveolar bone; these characteristics are consistent with a diagnosis of SMS. To date, this is the first case of SMS to be reported in China. *IFIH1* and *DDX58* have both been associated with SMS (Jang et al., 2015; Rutsch et al., 2015). In 2013, SMS patients with particular facial characteristics had been reported (broad forehead, a high anterior hairline, a thin upper vermillion, and a smooth philtrum) (Feigenbaum et al., 2013). Similarly, our proband also presented with unusual facial features. Facial characteristics may be a unique feature associated with SMS patients who possess gain-of-function mutations in the IF1H1 gene. We also recommend a genetic diagnosis for patient reported by Feigenbaum et al. (2013).

Aicardi-Goutières syndrome is a genetic form of encephalopathy. The neurological manifestations of AGS include dystonia, hypotonia, irritability, spastic quadriplegia, microcephaly, and severe developmental delay. Common extra-neurological manifestations include thrombocytopenia, lymphadenopathy, glaucoma hepatosplenomegaly, and chilblain lesions (Crow et al., 2015). Our proband demonstrated spastic paraplegia, multiple intracranial calcification, dystonia, psychomotor retardation, lymphadenopathy, pancytopenia, and autoimmunity. His phenotype was characteristic of AGS. Thrombocytopenia is common in patients with *IFIH1* gain-of-function mutations. However, to the best of our knowledge, pancytopenia is rare in cases with these mutations. Our proband showed hemoglobin, leukocyte, and platelet counts that were all below the normal values. The exact mechanism responsible for the pancytopenia caused by *IFIH1* gain-of-function mutation remains unclear; we can only speculate the relative contribution of the mutation to the clinical presentation. A persistently elevated serum level of IFN α may distort the immune system into an autoantibody-prone configuration (Van Eyck et al., 2015). The production of antibodies against hematopoietic cells, and the modifications made to the hematopoietic microenvironment by the chronic inflammation of AGS, may also contribute to hemopoietic failure. Another noteworthy condition is gastrointestinal disease. Our proband had experienced chronic diarrhea and provided positive fecal occult blood tests since he was young. This increased our suspicion of inflammatory bowel disease. Regretfully, the patient did not undergo colonoscopy to confirm this diagnosis.

Previous reports have described a pedigree with a c.1465G > A (p.Ala489Thr) mutation in *IFIH*1 (Bursztejn et al., 2015). The father of the proband in this case exhibited joint abnormalities and dental involvement that was typical of SMS, as well as the intracranial calcification that is typical of AGS; however, there was no evidence of neurological disease. In our proband, the phenotypic overlap between AGS and SMS was more obvious. Not only did he present with the severe neurological dysfunction that is typical of AGS, and the clinical features that are typical of SMS, he also had rare manifestations of the two syndromes. Table 2 shows a summary of the clinical features of the patient that overlap between AGS and SMS. IFIH1 has been associated with a number of autoimmune diseases, including diabetes (Nejentsev et al., 2009), psoriasis (Strange et al., 2010), systemic lupus erythematosus (Harley et al., 2008), and autoimmune thyroid disease (Sutherland et al., 2007). The precise reason for why a single gene associated with so many different autoimmune diseases with varying phenotypes remains unknown. One hypothesis is that the conformational changes created by different amino acid changes render MDA5 active to different endogenous dsRNAs. For example, the mutation of IFIH1 at residue Arg489 in patients with a phenotype that overlapped between SMS and AGS led to an impairment of ATP hydrolysis activity of MDA5. However, this effect was not evident in AGS patients exhibiting mutations at residues Arg720, Arg779, Gly495, or Asp393 (Rice et al., 2014; Bursztejn et al., 2015). In our case, the c.1465G > T mutation caused an amino acid substitution in the IFIH1 protein at residue Arg489. Just as Bursztejn described previously, the proband presented a phenotype that overlapped between AGS and SMS. To a certain extent, this involved genotype-phenotype correlations, thus indicating that these specific amino acid changes may be associated with a specific phenotype. Of particular note, even in a pedigree with same *IFIH1* mutation, there could still be significant variation in an individual's clinical phenotype (Rice et al., 2020). Furthermore, a single p.Arg822Gln mutation in the *IFIH1* gene has been detected separately in AGS and SMS (Buers et al., 2017). The variable phenotypes associated with IFIH1 gain-of-function mutations may be the consequence of the joint action of many factors, including genotype, epigenetic factors, and environmental factors.

**Table 2:** Clinical features of patient.

	**Patient 1 (Bursztejn et al., 2015)**	**Patient 2 (de Carvalho et al., 2017)**	**Patient 3 (Takeichi et‘al., 2018)**	**Our proband**
Mutation	c.1465G > A	c.992C > T	c.2561T > A	c.1465G > T
Assessment by interferon reporter assay	Yes	Yes	No	Yes
**Singleton-Merten syndrome**				
Abnormal dentition	+	+	+	+
Muscular weakness	–	+	–	+
Unusual face	–	+	–	+
Joint deformities	+	+	+	+
Aero-osteolysis	-	+	+	–
Alveolar bone resorption	–	–	–	+
Psoriasiform rash	–	+	–	–
**Aicardi-Goutières syndrome**				
Intracranial calcification	+	+	+	+
White matter hyperintensity	+	–	–	–
Developmental delay	–	–	+	+
Spastic dystonia	–	–	–	+
Spastic paraparesis	–	–	–	+
Hyperreflexia	–	–	–	+
Bulbar paralysis	–	–	–	+
Lymphadenopathy	–	–	–	+
Thrombocytopenia	–	–	–	+

*Compared with previously reported patients, the proband had more serious neurological dysfunctions and presented with a more obvious phenotypic overlap between AGS and SMS*.

AGS is a severe disease, urgently treatments are needed. Unfortunately, the proband was not correctly diagnosed until he was 30 years old. Before that, he only got some supportive and symptomatic therapies. Janus kinase (JAK) inhibitors are increasingly utilized in treatment of AGS, quite a few reports have showed encouraging results in AGS patients with the use of JAK inhibition (Kothur et al., 2018; McLellan et al., 2018). However, the major risks associated with JAK inhibitors among AGS patients were neutropenia, anemia and infection (Vanderver et al., 2020). Considering the patients' already existing blood system damage (pancytopenia) and other possible serious side effects of the drug, he was not treated with JAK inhibitors. He only received some rehabilitation treatments in our hospital.

In summary, we provide further evidence that SMS and AGS are associated with the same disease spectrum in the presence of *IFIH1* gain-of-function mutations, thus highlighting the genetic heterogeneity and expanded phenotype of gain-of-function mutations in the *IFIH1* gene.

## Data Availability Statement

The datasets for this article are not publicly available due to concerns regarding participant/patient anonymity. Requests to access the datasets should be directed to the corresponding author.

## Ethics Statement

The studies involving human participants were reviewed and approved by the Ethics Review Committee of Xiangya Hospital. The patients/participants provided their written informed consent to participate in this study. Written informed consent was obtained from the individual(s) for the publication of any potentially identifiable images or data included in this article.

## Author Contributions

WX drafted the manuscript. JF, HL, BX, and ZL revised the manuscript critically for important intellectual content. All authors contributed to the article and approved the submitted version.

## Conflict of Interest

The authors declare that the research was conducted in the absence of any commercial or financial relationships that could be construed as a potential conflict of interest. The reviewer MW declared a shared affiliation with several of the authors, WX, JF, HL, BX, and ZL to the handling editor at time of review.
