# Supplementary material for: Case Report: Aicardi-Goutières Syndrome and Singleton-Merten Syndrome Caused by a Gain-of-Function Mutation in IFIH1
Source: Front Genet. 2021 May 13;12:660953. doi: 10.3389/fgene.2021.660953 (PMC8155672; doi:10.3389/fgene.2021.660953)
Supplement: Supplementary file 1 [file Data_Sheet_1.docx]

**Method and Material**

Whole Exome Sequencing (WES)

5 ml blood was extracted from the patient, and then was send to RunningGene Company(Beijing, China), the DNA was extracted and the whole exome sequencing (WES) were performed following the standard protocols provided by RunningGene Company(Beijing, China).

qPCR assay:

Total RNA was extracted by TRIzol from peripheral blood lymphocyte of the patient and healthy control(matched with age and gender). Four micrograms of total RNA were subjected to reverse transcription, and 2 μL cDNA was used for PCR. Primer sequences were IFI27 F(5′- aatcgcctcgtcctccatag-3′) and IFI27 R (5′-tagaacctcgcaatgacagc-3′), IFI44L F(5′-gttgaaagatgcagccgtca-3′) and IF144L R(5′-aaacgacacaccagttgctc-3′), IFIT1 F(5′-aaaagcccacatttgaggtg-3′) and IFIT1 R (5′-gaaattcctgaaaccgacca-3′), ISG15 F(5′-tgtcggtgtcagagctgaag-3′) and ISG15 R (5′-gcccttgttattcctcacca-3′)，RSAD2 F （5′-cttttgctgggaagctcttg-3′）and RSAD2 R (5′-gtctcatctggccctctcag-3′), SIGLEC1 F (5′-actccacctttgcatggttc-3′）and SIGLEC1 R(5′-tagagcacacggagcatgac-3′), GAPDH F(5′- gagtcaacggatttggtcgt -3′) GAPDH R(5′- ttgattttggagggatctcg -3′). The primers were synthesized and purifiedat Qingke (Changsha, China). The RT reactions were performed using a cDNA synthesis kit (Vazyme, shanghai, China). Real-time PCR was performed using the ABI StepOnePlus Multicolor Real-Time PCR Detection System, and the assay was carried by the Sybr green kit(Vazyme, shanghai, China). The qPCR was start of 95℃ for 5 sec, followed by 40 cycles of 95°C for 15 sec and 60°C for 30 sec. A final melt-curve analysis (start of 95 °C for 15 sec, then 60 °C for 60 sec, followed by one cycles from 60 °C to 90 °C gradually increaseed 0.3 °C in each step for 60 sec. A final melt-curve analysis). The△Ct was normalized by housekeeping gene GAPDH. The relative expression of each gene in peripheral blood lymphocyte from the proband was normalized to controls and represented as a mean ± standard deviation.
