# Supplementary material for: Case Report: Aicardi-Goutières Syndrome and Singleton-Merten Syndrome Caused by a Gain-of-Function Mutation in IFIH1
Source: Front Genet. 2021 May 13;12:660953. doi: 10.3389/fgene.2021.660953 (PMC8155672; doi:10.3389/fgene.2021.660953)
Supplement: Supplementary file 2 [file Table_1.docx]

**Supplementary Table 1**

Overview and bioinformatic predictions of the novel IFIH1 mutation

| Variant | Protein change | Mutation type | gnomAD | ExAC | SIFT | PROVEAN | PolyPhen2 | Mutation Taster |
| --- | --- | --- | --- | --- | --- | --- | --- | --- |
| c.1465G>T | p.Ala489Ser | Missense | Not found | Not found | Deleterious | Deleterious | Probably  Damaging | Disease causing |

Bioinformatic predictions of the mutation, indicated that the mutation may influence the structure and function of the MDA5 protein.
